# The effect of domestication on post-conflict management: wolves reconcile while dogs avoid each other

**DOI:** 10.1098/rsos.171553

**Published:** 2018-07-04

**Authors:** Simona Cafazzo, Sarah Marshall-Pescini, Martina Lazzaroni, Zsófia Virányi, Friederike Range

**Affiliations:** 1 Wolf Science Center, Domestication Lab, Konrad Lorenz Institute of Ethology, University of Veterinary Medicine, Vienna, Austria; 2 Comparative Cognition, Messerli Research Institute, University of Veterinary Medicine Vienna, Medical University of Vienna and University of Vienna, Vienna, Austria

**Keywords:** conflict, captive wolves, cooperation, domestic dogs, reconciliation

## Abstract

Highly cooperative social species are expected to engage in frequent reconciliation following conflicts in order to maintain pack cohesiveness and preserve future cooperation. By contrast, in social species with low reliance on cooperation, reconciliation is expected to be less frequent. Here, we investigate the pattern of reconciliation in four captive wolf packs and four captive dog packs. We provide evidence for reconciliation in captive wolves, which are highly dependent on cooperation between pack members, while domestic dogs, which rely on conspecific cooperation less than wolves, avoided interacting with their partners after conflicts. Occurrence, intensity, latency, duration and initiation of wolf reconciliations appeared to vary as a consequence of a compromise between the costs (e.g. risk of further aggression) and the benefits (e.g. restoring relationship with opponents) of such interactions. Our results are in line with previous findings on various wolf packs living under different social and ecological conditions, suggesting that reconciliation is an important strategy for maintaining functional relationships and pack cohesiveness. However, current results on dogs are in contrast to the only other study showing that reconciliation can occur also in this species. Therefore, the occurrence of reconciliation in dogs may be influenced by social and environmental conditions more than in wolves. Which factors promote and modulate reconciliation in dogs needs to be further investigated.

## 1. Introduction

Social species inevitably experience within-group competition. These social conflicts may escalate into aggression leading to negative consequences, which range from the risk of injury to potential damage to social relationships and, as a consequence, disruption of group cohesion [1]. One way to cope with these negative effects is to repair the damaged relationships after conflicts. De Waal & van Roosmalen [2] first used the term ‘reconciliation' to refer to affiliative contacts between former opponents, which occur soon after the end of an agonistic conflict and may help to repair a relationship damaged by the aggressive encounter.

Since the original study documenting the occurrence of reconciliation in chimpanzees (*Pan troglodytes*) [2], a large number of studies have shown that reconciliation occurs in many other primate (reviewed by [3,4]) and non-primate species (e.g. *Capra hircus* [5]; *Tursiops* spp. [6]; *Crocuta crocuta* [7]; a review [4]; *Corvus frugilegus* [8]; *Canis lupus familiaris* [9]; *Canis lupus* [10,11]; *Corvus corax* [12]).

Whether reconciliation occurs is thought to be affected by a species' reliance on cooperation [1,4,13,14]: species characterized by high levels of cooperation are expected to engage in frequent reconciliation following conflicts (e.g. [15–17]) in order to re-establish peaceful relationships and maintain pack cohesiveness, potentially essential for future cooperative success [4,18]. In contrast, in species with low reliance on cooperation reconciliation is expected to be less frequent (e.g. [19–21]).

Wolves (*Canis lupus*) and domestic dogs (*Canis lupus familiaris*) are particularly good species in which to investigate and compare patterns of reconciliation. Wolves are highly dependent on cooperation because they rely on group hunting, cooperative breeding including alloparental care (e.g. [22–25]) and cooperative territorial defence (e.g. [26]). The cohesiveness and functionality of the packs are crucial for allowing wolves to successfully forage, raise pups and defend their territories [22,27,28]. Conversely, though free-ranging dogs have been observed to engage in joint territorial defence (e.g. [29–31]), alloparental care (e.g. [32–34]) and group hunting [35,36], they are mainly scavengers and exhibit a flexible and more promiscuous mating system [30,32,37,38] with pups being raised mostly by their mothers [32,38,39]. They appear to be ‘facultatively social’, e.g. forming close relationships with other dogs mainly if food abundance is high [30,40] and/or females are receptive [41]. Overall, such differences in their social ecologies demand less cooperation with conspecifics than wolves [42]. As such, we would expect wolves to show a higher reconciliatory tendency than dogs. Indeed, so far, reconciliation has been demonstrated in all wolf packs in which the phenomenon has been studied (two captive packs: European wolves [10]; Arctic wolves [43]; and two wild packs [11]), with the mean conciliatory tendency (i.e. the willingness to reconcile) ranging from 44% to 53%, which is relatively high compared with other species (e.g. hyenas: 16.6% [7]; chimpanzees: 16.3% [44]; bonobo: 35.6% [45]; ravens: 0.16% [12]; common marmosets: 31% [46]; tamarins: from 24.7% to 48.3% [47]; meerkats: 0% [18]). In the only study investigating conflict management in domestic dogs, reconciliation was shown to occur [9], and, although not reported by the authors, the mean conciliatory tendency based on the data published by Cools *et al*. ([9], table 4, p. 56) shows that, in line with our hypothesis, it is lower than that reported for wolves, ranging from 16.6% to 29.7%.

The purpose of our study was twofold. Our first aim was to investigate and compare the pattern of aggression and post-conflict (PC) interactions between former opponents in wolves and dogs living in similarly composed captive packs under identical conditions at the Wolf Science Center, Vienna, Austria. In line with previous findings on reconciliation in captive wolves and dogs and based on the observation that wolves, and under certain conditions also dogs, live in stable social packs, we expect conciliatory behaviours to occur in both species. Nevertheless, because dog packs rely on cooperation less than wolves, we expect to find a higher frequency of reconciliation in wolves than in dogs. We further investigated whether dogs and wolves might also use alternative PC strategies (e.g. [18]) by including an analysis of the patterns of proximity between opponents after conflicts.

In order to compare our results with previous findings and to extend our knowledge of reconciliation in both species, our second aim was to investigate the potential factors affecting reconciliation, in terms of its occurrence, latency, duration, intensity as well as its initiator. Previous studies on both dogs and wolves limited their analyses to factors affecting whether or not reconciliation occurred and who was more likely to initiate reconciliation. It has been shown in other species, however, that when and how reconciliation occurs, as well as who initiates reconciliation, may be influenced by variables related to the preceding conflict and to the quality of the two opponents' relationship (e.g. [48–53]).

In relation to the conflict characteristics, we included in our analyses (i) the intensity of the conflict (with versus without physical contact) and (ii) its context (whether it occurred in a feeding versus non-feeding situation).

We characterized the social relationship between opponents, based on daily observations of the animals, in terms of (i) the difference in their competitive abilities (measured by their rank distance and the asymmetry in the exchange of aggressive interactions) and (ii) the security of their relationship (measured by the asymmetry in the exchange of affiliative interactions).

In general, in line with previous findings in wolves and dogs, as well as in other species, we expect variation in reconciliation to be dependent on its potential costs and benefits as well as on the extent of the damage to the opponents' relationships (e.g. [10,11,43,52,53]). In particular, one of the main costs which opponents may incur when engaging in reconciliation is the risk of further aggression. In this respect, we expect rank distance and aggression asymmetry. In fact, the risk of escalation into further aggression is expected to be higher between opponents closer in rank and those showing a high symmetry in the exchange of aggressive behaviours (i.e. with more similar competitive abilities), which may be more likely to compete for a hierarchical position. When the risk of renewed aggression is high, opponents are expected to hesitate to engage in reconciliation. Therefore, we expect reconciliation to occur less frequently, later and with a shorter duration when conflicts involve opponents closer in rank and with higher aggression symmetry.

When competing over food resources, opponents need to find a balance between the benefits of restoring their relationships through reconciliation and the cost of a potential loss of time spent in accessing the food. Accordingly and in line with previous findings in other studies, we expect reconciliation to occur less frequently, sooner and with a shorter duration after conflicts over food (e.g. [43,52]) than after conflicts occurring in the absence of food.

In several species, including wolves and dogs, some evidence suggests that the damage to the opponents' relationship may be related to the intensity of the conflict, with higher intensity aggression causing higher levels of stress and tension in both opponents [9,43,52]. Therefore, we expect opponents to reconcile more often and potentially with a more intense interaction after a high-intensity conflict than after a low intensity one. However, as stress and tension decrease with time [54], aggression can be revived when reconciliation is initiated too quickly after the conflict [55]. Accordingly, reconciliation should be initiated later after higher intensity conflicts than after low intensity ones.

Although previous studies on wolves did not find an influence of the affiliative relationships on the occurrence of reconciliation, this was measured in terms of the frequency of affiliative behaviours exchanged by the dyad. However, another potentially more relevant measure of the social bond between opponents is the security of the relationship. This has been measured in previous studies in terms of the asymmetry of exchanged affiliative behaviours, assuming that those dyads with a high inequality in the exchange of affiliative behaviours are less secure [53,56,57]. Accordingly, we predict that opponents with a higher symmetry in the exchange of affiliative behaviours (i.e. more secure relationships) will engage earlier and longer in reconciliation than dyads with lower symmetric affiliative relationship (i.e. less secure relationships). Finally, according to previous results on both wolves and dogs, as well as on other species, we expect reconciliation to be initiated more often by victims than aggressors [11,43,58].

## 2. Material and methods

### 2.1 Subjects and study site

This study was conducted on four captive wolf packs and four captive dog packs (table 1 for pack composition) at the Wolf Science Center (www.wolfscience.at). The wolves that participated in this study originated from North America but were born in captivity, while the dogs were adopted from Hungarian shelters. All subjects were hand-raised in peer groups from the age of 10 days. They were bottle-fed and later hand-fed by humans, and had continuous access to humans in the first 5 months of their life. After 5 months they were introduced into the packs of adult animals, but still had daily social contact with humans during training and/or cognitive and behavioural experiments. All packs observed were composed of artificially assembled unrelated individuals (except for one wolf pack in which a sibling pair was present; see table 1 for details).

**Table 1.** Description of the packs studied.

pack	species	pack composition	observation period	total minutes of observation per pack per month
Kaspar	wolves	Kaspar ♂ Aragorn ♂ Tala ♀ Shima ♀ Chitto ♂	Jan 2013–Mar 2015	423
Geronimo	wolves	Geronimo ♂ Amarok ♂ Kenai ♂	Jan 2013–Nov 2014	454
Nanuk	wolves	Nanuk ♂ Yukon ♀ Una ♀	Apr 2013–Jul 2014	473
Wamblee	wolves	Wamblee ♂ Tatonga ♀	Jun 2013–Oct 2013	406
Rafiki	dogs	Rafiki ♂ Maisha ♂ Binti ♀ Hakima ♂	Jan 2013–Nov 2013	418
Kilio	dogs	Kilio ♂ Meru ♂ Nia ♀ Bashira ♀	Jan 2013–Jul 2013	335
Nuru	dogs	Nuru ♂ Layla ♀ Zuri ♀	Jan 2013–Mar 2014	431
Asali	dogs	Asali ♂ Bora ♀	Jan 2013–Nov 2014	470

Packs live in large enclosures (4000–8000 m^2^ for wolves, 3000–4000 m^2^ for dogs) equipped with trees, bushes and shelters. Water for drinking is permanently available. Animals receive a diet of meat, fruits, milk products and dry food. During the first months of their lives, they were fed several times per day, which was slowly reduced to being fed major meals daily (dogs) or only two or three times per week (wolves) according to their natural rhythm.

### 2.2 Data collection

Data were collected from January 2013 until March 2015. All packs were observed over 2 days per week for approximately 1 h a day, either in the morning or in the afternoon (table 1) for a total of 927.13 h of observation (523.87 h for wolf packs and 403.26 for dog packs).

To ensure that the relationships between the animals were characterized based on data independent of the PC behaviour, we adopted two sampling methods: (i) we carried out ‘focal animal' sampling of all individuals in the pack, focusing on the social behaviours exchanged with all other pack members, and on the basis of this dataset calculated the relationship indices used in the analyses, and (ii) to collect data on the PC behaviours we adopted ‘behavioural’ sampling methods [59], video-recording the victim of a conflict.

Focal animal sampling lasted 10 min and was carried out using the program Pocket Observer v. 2.1.23.2 (Noldus Information Technology) on a hand-held device (Samsung Galaxy Note 2). We recorded all occurrences of the focal animal's aggressive, dominant, submissive (used to assess the hierarchical rank) and affiliative interactions and towards whom they were directed (see electronic supplementary material, table S1) (for a total of 421.83 h of observation; 17.17 ± 13.99 mean ± s.d. per subject).

Behavioural sampling sessions for PC behaviour were started when an aggressive encounter began and ended 10 min after it ended. During this time we continuously filmed the victim as the focal individual for a 10 min PC period. Control observations (matched controls, MCs) took place the next possible day at the same time as the original PC, on the same focal animal, in the absence of agonistic interactions occurring during the 10 min before the beginning of the MC [60,61]. For each aggressive encounter we recorded: (i) the opponents, (ii) the context (presence or absence of food), (iii) the outcome of conflict (decided or undecided), and (iv) aggressive behavioural patterns (see electronic supplementary material, table S2). Decided conflicts were defined as those conflicts in which one individual performed an aggressive behaviour and the other responded with a submissive behaviour. Cases in which both were aggressive or in which the recipients of the aggression did not show any clear submissive behaviour or answered by showing dominant behaviours were defined as undecided. For undecided conflicts, we defined as the aggressor the wolf/dog that was first seen being aggressive.

Aggressive behaviours were then categorized according to two levels of aggressive intensity: level 1: low intensity—aggressions without physical contact (threat, chase, jaw spar and snap); and level 2: high intensity—aggressions with physical contact (attack, knock down, stand over aggressive, pin, fight and bite) (see the electronic supplementary material, table S2, for detailed descriptions of the behaviours).

Finally, video records of the PC and MC observations were analysed using the software Solomon Coder^®^ (András Péter). For both PCs and MCs we recorded (i) starting time (min), (ii) the minute of the first affiliative behaviour, (iii) type and duration of the affiliative interaction, (iv) the initiator of the affiliative behaviour, and (v) the duration of the time spent by the victim in close proximity to the aggressor (see the electronic supplementary material, table S2, for a detailed description of the behaviours).

### 2.3. Inter-observer reliability

Forty-two PC–MC pairs (i.e. 23.7% of the total observations) were video-recorded and subsequently analysed independently by the two main observers (S.C. and M.L.) to obtain inter-observer reliability coding. We calculated a κ coefficient [62,63] for all relevant behaviours (affiliative and proximity interactions, *k* = 0.81 and *k* = 0.74, respectively), timing (*k* = 0.76) and partner identities (*k* = 0.97). The overall averaged κ coefficient was *k* = 0.82.

### 2.4. Data analyses

#### 2.4.1. Measure of relationship quality

In order to characterize the relationship quality between aggressor and victim within the packs, we used data from our focal animal observations to calculate the following behavioural variables: (i) the asymmetry in the exchange of affiliative and (ii) aggressive behaviours and (iii) the dyadic rank distance.

For (i) and (ii) we calculated the asymmetry in the exchange of affiliative (AFFav) and aggressive (AGGav) behaviours for each dyad according to the following formula (adapted from [53]):

$$\text{AFFav( AGGav) }=\frac{Ba\to Bv}{Ba↔Bv}-\frac{Bv\to Ba}{Ba↔Bv},$$

where *Ba *→ *Bv* is the total number of behaviours that *the aggressor* directed at *the victim* during the focal observations, *Bv → Ba* is the amount of behaviours that *the victim* directed at *the aggressor*, and *Ba *↔ *Bv* is the total amount of behaviour exchanged between *aggressor* and *victim*.

A value close to ‘0' means that the two animals exchange the behaviours equally frequently, that is, their relationship is symmetric. A value close to ‘−1' means that the victim shows more behaviours towards the aggressor than vice versa, whereas a value close to ‘+1' means that the reverse is true.

The asymmetry in the exchange of *affiliative behaviours* has been used in other species as a measure of the security of the relationships [53,56,57]; in particular, dyads showing a high asymmetry are assumed to have a less secure relationship. Asymmetry in the exchange of *aggressive behaviours* is instead considered a measure of the differences in competitive abilities between two subjects; with dyads showing a high asymmetry with highly diverse competitive skills.

For (iii), for each individual we calculated the David's score [64] based on the direction and frequency of submissive behaviours recorded during the focal observations. Then, we used the value of the difference between the individual scores to represent the dyadic rank distance (see the electronic supplementary material for more details).

#### 2.4.2. Measures of reconciliation

To investigate the occurrence of reconciliation, we used two kinds of analyses and took only those dyads that were involved in at least three conflicts into consideration (for a total of 15 dyads and 175 conflicts for wolves and five dyads and 26 conflicts for dogs). First, to compare the timing of the first affiliative interaction between former opponents during one PC with that during the corresponding MC, we followed de Waal & Yoshihara's [60] PC–MC method. A PC–MC pair was labelled ‘attracted' if the former opponents affiliated only in the PC or earlier in the PC than in the MC. Similarly, a PC–MC pair was labelled ‘dispersed' if the former opponents affiliated only in the MC, or earlier in the MC than in the PC. When affiliative contacts occurred during the same minute in the PC and the MC, or no contact occurred in either the PC or the MC, the PC–MC pair was labelled ‘neutral'. For each individual, we compared the proportion of ‘attracted' PC–MC pairs with the proportion of ‘dispersed' pairs by using a Wilcoxon matched-pair signed-ranks test (corrected for ties [65]). Second, in order to establish the specific time frame in which an affiliative behaviour is more likely to function as a ‘reconciliation' event, we used the ‘time rule' method (compares the frequency of PC affiliative interactions between opponents occurring within a specified timeframe, i.e. the 10 min PC focal, in PC versus MC periods; see [3], pp. 22 and [66]). Following this method, we determined for each PC and MC observation the minute in which the first affiliative contact between opponents occurred. Next, we compared the distribution of first PC events with first MC events using the Kolmogorov–Smirnov test. If this test produced a significant result, we ran a Wilcoxon matched-pair signed-ranks test comparing individual PC and MC scores within the time period in which the PC distribution differed from that for the MC. In this way we checked the generality of this phenomenon at the individual level.

Overall, reconciliation is considered to occur when following both the PC–MC method and the ‘time rule' method, former opponents made friendly contacts earlier in PCs than in MCs.

Following Veenema *et al*. [67], to calculate each individual's corrected conciliatory tendency (CCT), we followed the formula: attracted minus dispersed pairs divided by the total number of PC–MC pairs (attracted + dispersed + neutral pairs). Individual CCTs were then used to determine the mean group CCT for species comparison.

All non-parametric tests (two-tailed) were conducted in STATISTICA 7.1 edition (StatSoft Italy s.r.l. 2005). The probability level for rejection of the null hypothesis was set at 0.05.

#### 2.4.3. Dog–wolf comparison of aggression and proximity patterns

In order to achieve our first aim, that is, to compare dogs and wolves in regard to their proportion and intensity of aggression, the occurrence of reconciliation and proximity between opponents after conflicts, we used the Mann–Whitney test (two-tailed) conducted in STATISTICA 7.1 edition (StatSoft Italy s.r.l. 2005). The probability level for rejection of the null hypothesis was set at 0.05.

#### 2.4.4. Exploring variation in reconciliation

##### 2.4.4.1. Measures of variation in reconciliation

A reconciliation event, defined as either the victim or the aggressor displaying an affiliative behaviour towards its opponent, was further characterized in a number of ways: (i) the latency of its occurrence, that is, the time elapsed between the end of the conflict and the beginning of the first PC affiliative interaction, (ii) the duration of the reconciliation event, that is, the time elapsed from the start of the first affiliative behaviour to the end of the last consecutive affiliative behaviour displayed by the initiator of reconciliation, and (iii) the intensity of the reconciliation event based on the affiliative intensity displayed: low-intensity reconciliation—only one affiliative behavioural pattern was displayed without involving any physical contact between the opponents (e.g. approach friendly and stand friendly)—and high-intensity reconciliation—one or more affiliative behavioural patterns were displayed with at least one of these involving physical contact between opponents (e.g. nose touch, muzzle-licking and body rubbing), (see the electronic supplementary material, table S2, for a detailed description). Both the duration and intensity of reconciliation were, to some extent, dependent on the number of affiliative behavioural patterns displayed during the reconciliatory interaction. However, high-intensity reconciliations did not last significantly longer than low-intensity ones (Mann–Whitney test: *U_39,30_* = 518.5, *Z*_adj_ = 0.1, *p* = 0.42; mean durations ± s.d.: high-intensity reconciliations, 7.33 ± 8.81 s; low-intensity reconciliations, 9.33 ± 13.17 s), suggesting that the duration and intensity of reconciliation were independent from each other.

##### 2.4.4.2. Variables potentially affecting variation in reconciliation: test models

Table 2 outlines the variables considered as potential predictors of the occurrence, latency, duration and intensity as well as the initiator (aggressor versus victim) of reconciliation. To test which factor may affect the occurrence of reconciliation (model 1), its initiator (model 2) and its intensity (model 3), we ran generalized mixed-effect models (GLMMs) with a binomial distribution. To detect which independent variables may affect the latency (model 4) and duration (model 5) of reconciliation we ran linear mixed-effect models (LMMs). Models 2, 3, 4 and 5 were restricted to ‘attracted' PC–MC pairs (for a total of 69 pairs), thus only PCs in which reconciliation occurred (i.e. all PCs in which a friendly contact occurred earlier than in MCs). Model assumptions (i.e. over-dispersion for GLMMs and normally distributed residuals and homogeneity of variances for LMMs) were met. We included, as random factors, the identity of the victim for each dyad nested into dyad and pack identity. Random factors allow the inclusion in the model of multiple data collected on the same victim or dyad/pack, thus controlling for non-independence of the samples [68].

**Table 2.** Name and description of the variables considered in the test models. In Model 1, we analysed 175 PC–MC pairs for a total of 15 dyads. Models 2, 3, 4 and 5 were restricted to ‘attracted' PC–MC pairs, for a total of 69 pairs and 13 dyads.

variable	type and description
*response variable*	
Model 1. occurrence of reconciliation	categorical (yes–no)
Model 2. initiator of reconciliation	categorical (victim–aggressor)
Model 3. intensity of reconciliation	categorical (low intensity–high intensity)
Model 4. latency of reconciliation	continuous (the seconds from the end of a conflict to the occurrence of reconciliation)
Model 5. duration of reconciliation	continuous (the seconds from the beginning of the first affiliative behaviour to the last one)
*independent variable* (for all models)	
1. rank distance	continuous (differences of individual David' scores)
2. degree of affiliation symmetry	continuous (AFFav)^a^
3. degree of aggression symmetry	continuous (AGGav)^a^
4. conflict intensity	categorical (high intensity–low intensity)
5. context of the conflict	categorical (feeding context–non-feeding context)

^a^See text for further details.

For all analyses to evaluate and compare different models, we used the Akaike information criterion, corrected for small sample sizes (AICc), and adopted a model-averaging approach (using the package MuMIn in R), allowing us to evaluate also the ‘relative importance' of each variable (RVI) across models [69,70].

All model analyses were performed using R v. 3.3.3. We implemented linear mixed-effects models using the ‘lmer' function of the ‘lmerTest' package [71] and generalized mixed-effects models using the ‘glmer' function in the ‘lme4'package [72]. All data are provided in the electronic supplementary material.

## 3. Results

### 3.1. Pattern of aggressions in wolf and dog packs

In wolf packs, we recorded 419 aggressive interactions (0.80 aggressions per observation hour) and a total of 177 PC–MC pairs^1^ involving 12 subjects as victim (one adult male, Nanuk, was never recorded as a conflict victim). The number of PCs per wolf ranged from 1 to 47 with an average number of conflicts per focal of 13.62 ± 14.07. Of the total 177 PC episodes, 45 occurred in the food context and 132 outside of it. Furthermore, most of the conflicts had a clear outcome (155 were decided and only 22 were undecided). Finally, 59.3% (*N* = 105) of conflicts were characterized by aggression of high intensity and 40.7% (*N* = 72) by low-intensity aggression.

**Figure 1.**
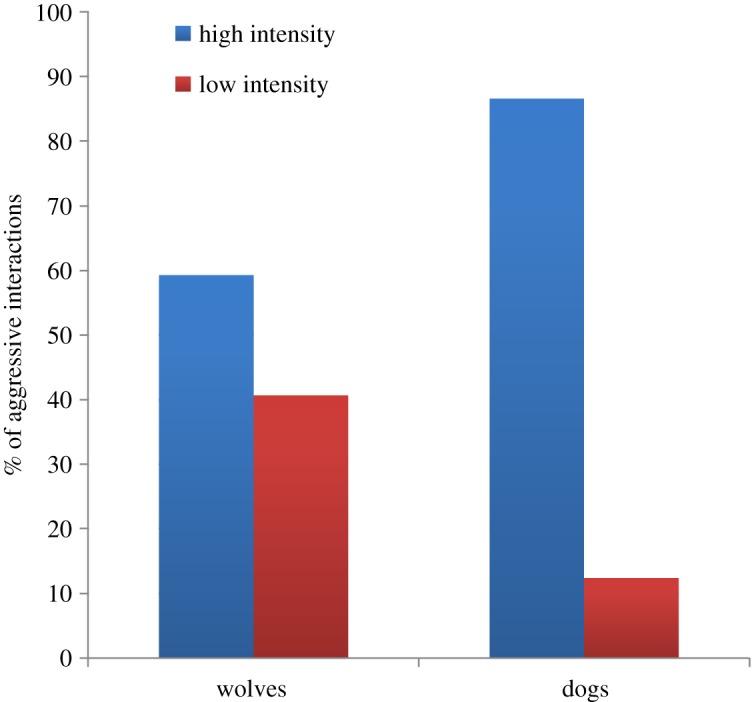
Pattern of aggressive interactions in wolves and dogs.

In dogs, the number of aggressive interactions recorded was much lower than in wolves. In fact, during the observation period, we recorded only 55 aggressive interactions (0.14 aggressions per observation hour) and a total of 30 PC–MC pairs involving eight subjects as victim (four adult male dogs, Rafiki, Kilio, Nuru and Asali, and one young female, Nia, were never recorded as a conflict victim). The number of PCs per dog ranged from one to seven with an average number of conflicts per focal of 3.75 ± 2.44. Of the total 30 PC episodes, six occurred in the food context and 24 outside of it. As for wolves, most of the conflicts had a clear outcome (27 were decided and only three were undecided), but, in contrast to wolves, the higher proportion of conflicts were characterized by high-intensity aggressions (86.6% (*N* = 28) were of high intensity while only 12.4% (*N* = 2) were of low intensity). In fact, we found that dog dyads showed significantly more high-intensity conflicts than wolf dyads (Mann–Whitney test: *U*_15,8_ = 11, *Z*_adj_ = −3.23, *p* = 0.0008; figure 1).

### 3.2. Does reconciliation occur in wolves and dogs?

**Figure 2.**
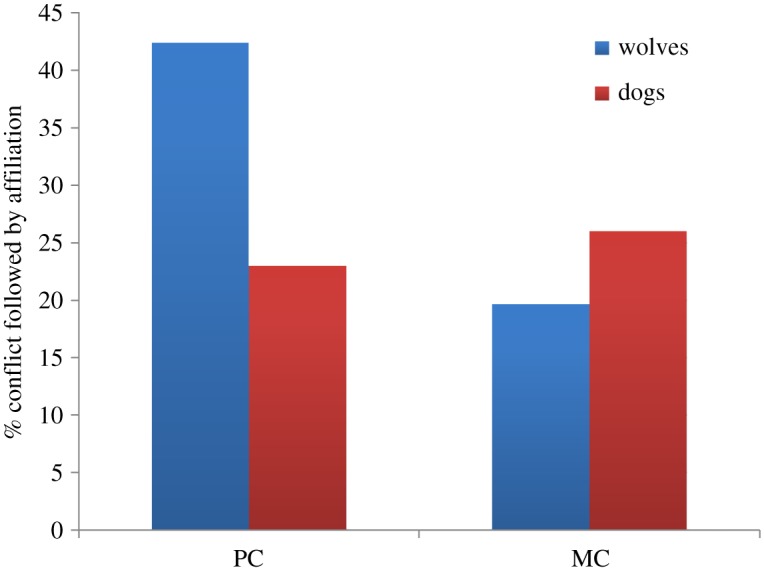
Percentage of PCs and MCs followed by affiliation for both wolves and dogs.

**Figure 3.**
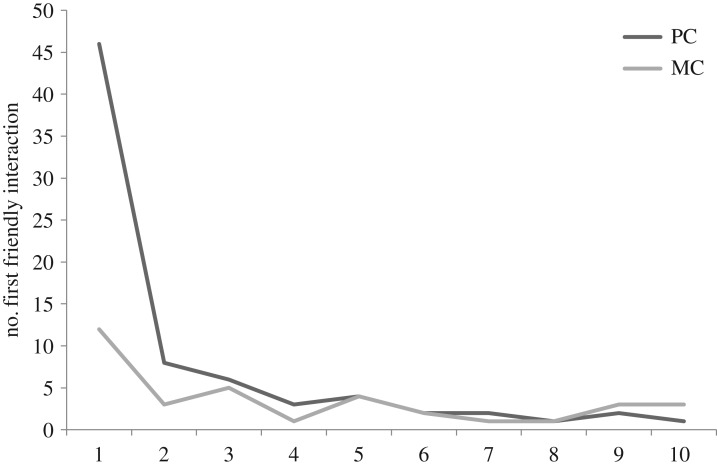
Temporal distribution of first affiliative interactions in PC and MC periods.

Following the PC–MC method, wolf victims and aggressors were significantly more likely to interact affiliatively with former opponents in PCs than MCs (42.4% versus 19.8%; figure 2). In fact, in the wolves we found significantly more attracted than dispersed PC–MC pairs (Wilcoxon signed-ranks test: *Z* = 2.55, *n* = 10, *p* = 0.01). This indicates that the majority of affiliative contacts between the opponents occurred earlier in PC than in MC periods. The time rule method confirmed this effect. The temporal distribution of first affiliative interactions between former opponents in the PCs was significantly different from that in the MCs (Kolmogorov–Smirnov test: *D* = 0.01, *p* < 0.001; figure 3).

The greatest difference in the cumulative distributions was during the first minute. Importantly, this result is not due to a few individuals, as most wolf victims were involved in affiliation with former aggressors in the first minute of more PCs than in the first minute of their MCs (Wilcoxon signed-ranks test: *Z* = 1.96, *n* = 10, *p* = 0.04). The mean CCT of all focal individuals was 22.46%, ranging from −25.00% to 63.16% (table 3). Based on the two methods outlined above, we could consider 71 conflicts out of a total of 177 as reconciled.

**Table 3.** Corrected conciliatory tendencies (CCTs) in wolves, number of attracted, dispersed and neutral pairs for each victim. Only victims involved in more than three conflicts were included (thus, two subjects, Wamblee and Kaspar, who were involved in one and two conflicts, respectively, were not included in the analysis). Group CCT%, the mean CCTs of the pack; s.e., standard error.

focal/victim	attracted	dispersed	neutral	total	CCT%
Aragorn	12	4	8	24	33.33%
Tala	13	1	5	19	63.16%
Shima	5	4	24	33	3.03%
Chitto	25	11	11	47	29.79%
Geronimo	1	0	2	3	33.33%
Amarok	3	2	7	12	08.33%
Kenai	2	0	10	12	16.67%
Yukon	3	1	2	6	33.33%
Una	5	1	8	14	28.57%
Tatonga	0	1	3	4	−25.00%
**group CCT%**					**22**.**46%**
**s.e.**					**0**.**07%**

**Table 4.** Number of attracted, dispersed and neutral pairs for each dog victim.

focal/victim	attracted	dispersed	neutral	total
Maisha	0	0	7	7
Binti	0	0	4	4
Hakima	0	0	1	1
Meru	2	1	4	7
Bashira	0	0	2	2
Bora	2	2	1	5
Layla	1	0	0	1
Zuri	1	2	0	3

Contrary to wolves, dog victims and aggressors were not more likely to interact affiliatively with former opponents in PCs than in MCs (23.3% versus 26.7%; figure 2). Although we had only 30 PC–MC pairs, we found no difference between the number of attracted and dispersed PC–MC pairs (Wilcoxon signed-ranks test: *Z* = 0.54, *n* = 8, *p* = 0.59; table 4).

### 3.3. Patterns of proximity during post-conflict observations in wolves and dogs

The analyses of proximity revealed that, after a conflict (i.e. during PC periods), dog victims spent significantly less time in close proximity to their aggressor than wolves (Mann–Whitney test: *U*_15,8_ = 13.5, *Z*_adj_ = 3.02, *p* = 0.001), while no difference between the two species emerged during MC periods (Mann–Whitney test: *U*_15,8_ = 55, *Z*_adj_ = −0.32, *p* = 0.78). This result indicates that dog opponents do not seek an opportunity to reconcile but rather choose to avoid each other. Furthermore, during PCs, the occurrence of reconciliation did not influence the proximity between wolf opponents because there was no difference in the duration of proximity between reconciled versus un-reconciled PCs (Mann–Whitney test: *U*_106,71_ = 3244, *Z*_adj_ = −1.70, *p* = 0.13). Therefore, the finding that, after conflicts, wolf opponents spent more time in close proximity than dogs is not just a consequence of reconciliation.

Since we could not find any evidence of the occurrence of reconciliation in dogs, data on PC behaviour of dogs were not further analysed.

### 3.4. Variation of reconciliation in wolves

#### 3.4.1. What factors affect the occurrence of post-conflict friendly interactions (model 1)?

We found an effect of the rank distance between opponents, context and intensity of aggression with a higher likelihood of reconciliation occurring when the victim and the aggressor were more distant in rank, when the conflict happened without food present and after high-intensity aggression.

In particular, of the 45 conflicts occurring when food was available, 5.08% (*n* = 9) were followed by a friendly interaction, whereas of the 130 conflicts occurring in the non-food context 46.92% (*n* = 61) were followed by a friendly interaction. In the same way, of the 104 conflicts characterized by high-intensity aggression, 48 were followed by a friendly interaction (46.15%), whereas of the 71 conflicts with low-intensity aggression only 22 were followed by a friendly interaction (30.99%).

The asymmetry in the exchange of affiliative and aggressive behaviours had no effect on the likelihood of a friendly interaction occurring. Model comparison is shown in table 5 while estimated effect sizes, relative variable importance and *p*-values are shown in table 6.

**Table 5.** Model comparison for factors affecting the occurrence of post-conflict friendly interactions (model 1). Models are reported up to three AICc points from the best model.

	d.f.	logLik	AICc	delta	weight
context, conflict intensity and rank distance	6	−101.64	215.79	0.00	0.34
context, conflict intensity, rank distance and degree of aggression symmetry	7	−101.56	217.79	2.00	0.12
context, conflict intensity, rank distance and degree of affiliation symmetry	7	−101.62	217.91	2.12	0.12
context, conflict intensity and degree of aggression symmetry	6	−103.07	218.64	2.85	0.08

**Table 6.** Estimated effect size, adjusted standard error (s.e.), *Z*-value and relative variable importance (RVI) estimated by a generalized linear mixed model to determine the effects of each variable on the occurrence of reconciliation (model 1).

	effect size	adjusted s.e.	*Z*-value	*p*	RVI
context of conflict	1.59093	0.51353	3.098	0.00195	0.99
conflict intensity	−0.83539	0.38686	2.159	0.03082	0.82
rank distance	0.11886	0.05447	2.182	0.02911	0.77
degree of aggression symmetry	0.80278	1.08487	0.740	0.45932	0.34
degree of affiliation symmetry	−0.35695	1.21023	0.295	0.76804	0.26

#### 3.4.2. What factors affect the initiator identity of post-conflict friendly interactions (model 2)?

When reconciliation occurred between wolf opponents, it was more likely to have been initiated by the victim (58) than the aggressor (13) (Mann–Whitney test: *U*_71,71_ = 958.5, *Z*_adj_* *= 7.36, *p* < 0.000001). In the majority of cases, the victim was also the subordinate (56) individual (Mann–Whitney test: *U*_71,71_ = 923, *Z*_adj_* *= 7.53, *p* < 0.000001). Only the context of the conflict had a significant effect on the identity of the initiator of PC friendly interaction. In particular, the majority of PC affiliative interactions initiated by the victim occurred in the non-feeding context (victims: 53; aggressors: 9), while both victim and aggressor were equally responsible for the initiation of reconciliation in the feeding context (victims: 5; aggressors: 4). Intensity of aggression as well as the asymmetry in the exchange of affiliative and aggressive behaviours had no effect on the initiator identity of friendly interactions. Model comparison is shown in table 7, while estimated effect sizes, relative variable importance and *p*-values are shown in table 8.

**Table 7.** Model comparison for factors affecting the initiator identity of post-conflict friendly interactions (model 2). Models are reported up to 2 AICc points from the best model.

	d.f.	logLik	AICc	delta	weight
context and rank distance	5	−29.15	69.26	0.00	0.25
context, conflict intensity and rank distance	6	−28.97	71.30	2.04	0.09

**Table 8.** Estimated effect size, adjusted standard error (s.e.), *Z*-value and RVI estimated by a generalized linear mixed model to determine the effects of each variable on the initiator identity of post-conflict friendly interactions (model 2).

	effect size	adjusted s.e.	*Z*-value	*p*	RVI
context of conflict	2.02815	0.94765	2.140	0.0323	0.80
conflict intensity	0.33099	0.78980	0.419	0.6752	0.26
rank distance	0.12551	0.07089	1.771	0.0766	0.68
degree of aggression symmetry	0.26218	0.96416	0.272	0.7857	0.25
degree of affiliation symmetry	−0.86165	1.82049	0.473	0.6360	0.25

#### 3.4.3. What factors affect the intensity of post-conflict friendly interactions (model 3)?

High-intensity PC affiliative interactions occurred more frequently than low-intensity ones (high intensity: 56.52%; low intensity: 43.48%). The intensity of PC friendly interaction was affected by context, intensity of aggression and asymmetry in the exchange of aggressive behaviours. In fact, conflicts in the non-feeding context were followed more often by highly intense friendly interactions (*N* = 38) than low-intensity ones (*N* = 22), whereas the reverse was true in the feeding context (high intensity: 1; low intensity: 8). Furthermore, high-intensity, but not low-intensity, reconciliations occurred more often after high-intensity (*N* = 32) than low-intensity aggressive interactions (*N* = 7). Finally, reconciliation between former opponents was more frequently of high intensity for those dyads with higher aggression asymmetry, thus in dyads in which the aggressor showed a higher frequency of aggressive behaviours towards the victim than vice versa.

Rank distance between opponents and asymmetry in the exchange of affiliative behaviours had no effect on the intensity of friendly interactions. Model comparison is shown in table 9, while estimated effect sizes, relative variable importance and *p*-values are shown in table 10.

**Table 9.** Model comparison for factors affecting the intensity of post-conflict friendly interactions (model 3). Models are reported up to three AICc points from the best model.

	d.f.	logLik	AICc	delta	weight
context, conflict intensity and degree of aggression symmetry	6	−36.60	86.55	0.00	0.28
context, conflict intensity, degree of aggression symmetry and degree of affiliation symmetry	7	−35.76	87.36	0.81	0.19
context, conflict intensity, rank distance and degree of aggression symmetry	7	−36.40	88.64	2.09	0.10
context, conflict intensity, rank distance, degree of aggression symmetry and degree of affiliation symmetry	8	−35.29	88.98	2.44	0.08

**Table 10.** Estimated effect size, adjusted standard error (s.e.), *Z*-value and RVI estimated by a generalized linear mixed model to determine the effects of each variable on the intensity of reconciliation (model 3).

	effect size	adjusted s.e.	*Z*-value	*p*	RVI
context of conflict	2.69049	1.17121	2.297	0.0216	0.95
conflict intensity	−1.34395	0.63963	2.101	0.0356	0.76
rank distance	0.04749	0.06058	0.784	0.4330	0.30
degree of aggression symmetry	2.61194	1.13596	2.299	0.0215	0.92
degree of affiliation symmetry	2.14532	1.68810	1.271	0.2038	0.42

#### 3.4.4. What factors affect the latency of post-conflict friendly interactions (model 4)?

Reconciliation mainly took place within the first 2 min from the end of a conflict although its latency was highly variable, ranging from 0 to 582 s (mean 99.4 ± 145.8 s between the end of a conflict and the occurrence of reconciliation ± s.d.). None of the predictor variables had an effect on the timing of reconciliation. Model comparison is shown in table 11, while estimated effect sizes, relative variable importance and *p*-values are shown in table 12.

**Table 11.** Model comparison for factors affecting the latency of post-conflict friendly interactions (model 4). Models are reported up to 2 AICc points from the best model.

	d.f.	logLik	AICc	delta	weight
context	5	−439.59	890.12	0.00	0.12
context and conflict intensity	6	−438.68	890.71	0.59	0.09
null model	4	−441.19	891.00	0.87	0.08
context and degree of affiliation symmetry	6	−439.39	892.13	2.00	0.05

**Table 12.** Estimated effect size, adjusted standard error (s.e.), *Z*-value and RVI estimated by a generalized linear mixed model to determine the effects of each variable on the latency of reconciliation (model 4).

	effect size	adjusted s.e.	*Z*-value	*p*	RVI
context of conflict	−94.710	53.449	1.772	0.0764	0.56
conflict intensity	−44.415	37.932	1.171	0.2416	0.38
rank distance	−2.504	3.903	0.642	0.5212	0.28
degree of aggression symmetry	78.631	61.198	1.285	0.1988	0.39
degree of affiliation symmetry	98.217	99.905	0.983	0.3256	0.32

#### 3.4.5. What factors affect the duration of post-conflict friendly interactions (model 5)?

The duration of reconciliation was highly variable, ranging from 0.6 to 65 s (8.2 ± 10.8, mean seconds ± s.d.). We found an effect of asymmetry in the exchange of affiliative behaviours with reconciliation between former opponents lasting longer for those dyads in which the aggressor showed more affiliative behaviours towards the victim than vice versa.

All other variables had no effect on the timing of reconciliation. Model comparison is shown in table 13, while estimated effect sizes, relative variable importance and *p*-values are shown in table 14.

**Table 13.** Model comparison for factors affecting the duration of post-conflict friendly interactions (model 5). Models are reported up to three AICc points from the best model.

	d.f.	logLik	AICc	delta	weight
context, conflict intensity, degree of aggression symmetry and degree of affiliation symmetry	8	−247.73	513.87	0.00	0.39
conflict intensity, degree of aggression symmetry and degree of affiliation symmetry	7	−250.03	515.89	2.03	0.14
context, conflict intensity and degree of affiliation symmetry	7	−250.34	516.51	2.65	0.10
context, degree of aggression symmetry and degree of affiliation symmetry	7	−250.48	516.80	2.93	0.09

**Table 14.** Estimated effect size, adjusted standard error (s.e.), *Z*-value and RVI estimated by a generalized linear mixed model to determine the effects of each variable on the duration of reconciliation (model 5).

	effect size	adjusted s.e.	*Z*-value	*p*	RVI
context of conflict	0.319683	4.035359	0.079	0.9369	0.74
conflict intensity	3.595657	2.883278	1.247	0.2124	0.81
rank distance	−0.001679	0.321753	0.005	0.9958	0.17
degree of aggression symmetry	3.104918	4.429229	0.701	0.4833	0.79
degree of affiliation symmetry	17.146720	7.361661	2.329	0.0198	0.98

## 4. Discussion

### 4.1. Comparing patterns of aggression and post-conflict behaviours in wolves and dogs

Our results highlight a number of interesting differences between dogs and wolves in both their conflict and PC behaviours. Dogs showed significantly lower rates of aggression than wolves; however, when aggression did occur it was of higher intensity (i.e. involving physical contact). Interestingly, these results are in line with early studies of similarly raised packs of wolves and dogs, which lead to the suggestion that ritualized aggression in domestic dog may have been progressively lost during the course of domestication [73–75].

After conflicts, wolves were more likely to reconcile, whereas dogs appeared to adopt an avoidance strategy, because dogs but not wolves spent more time away from their partners after a conflict than in control periods. This is also reflected in the animal management at the Wolf Science Center as we had to remove some dogs from the packs because conflicts between dogs escalated into severe aggression more often than conflicts between wolves. During the study, three dogs had to be removed from the packs, whereas no wolf was taken out. While this may lead to an underestimation of the frequency of aggressive interactions/conflicts that occurred in dogs, it does not affect the estimation of the tendency to reconcile because this measure is proportional to the conflicts that occurred in the respective species.

In wolves, the number of attracted pairs was significantly higher than the number of dispersed pairs, indicating that former opponents show a higher affiliative tendency in PC situations than without a preceding conflict. Supporting this suggestion, reconciliation occurred mainly in the first minute. The mean conciliatory tendency of our wolf packs (22.46%) was lower than that found in two wild packs of Canadian timber wolves (CCT = 44.1% [11]), and in captive families of European (CCT = 53.2% [10]) and Arctic wolves (CCT = 46.87% [43]). However, our packs differ from all previous studied packs in two respects. First, our packs were smaller in size (3.5 ± 1.26 individuals) than the captive pack of European wolves (nine individuals) and Arctic wolves (19 individuals). Second, our packs were composed of artificially assembled unrelated individuals, whereas all the other packs studied were families composed of the breeding pair and their offspring of the previous year (with the exception of the European wolf pack in which only the alpha-male was present together with the offspring [10]). While the number of available social partners does not seem to influence the occurrence and frequency of reconciliation (chimpanzees [44]; wolves: nine individuals [10]; 19 individuals [43]), in a number of species reconciliation is more frequent among related than unrelated individuals (the kinship hypothesis [12,76–78]). Considering that wild wolf packs are composed of related members that cooperate in a wide variety of activities, relatedness is likely to be an important aspect of wolves' sociality. As such, in the current study pack composition is more likely than pack size to account for the low level of CCT.

In contrast to wolves, in our packs of domestic dogs, we found no evidence of reconciliation after aggressive encounters. These results are in contrast with those found in the only other study investigating reconciliation in three captive groups of dogs [9]. The study differed from ours in a number of ways—two in particular, which are thought to be crucial as regards the contrasting results, are (i) the relatedness between pack members and (ii) the behaviours considered as aggressive and affiliative interactions.

Our dog packs were not composed of related individuals (with the only exception of two siblings, Nuru and Zuri, which were both members of the same pack), while several dogs in the study of Cools *et al*. [9] were related to each other. Relatedness is known to affect reconciliation rates [12,76–78], and indeed Cools *et al.* also found that reconciliation occurred more in ‘familiar' than in ‘unfamiliar' dogs (where familiarity and relatedness overlapped to a large extent in the study). It is, therefore, possible that reconciliation rates in our pack were particularly low because no such relatedness bonds were present. Second, the ethogram used by Cools *et al*. [9] was different from the one used in the current study. In particular, they considered ‘directed barking' and ‘anogenital sniffing' as light aggression and affiliation, respectively. Actually, both behavioural patterns may have other functions (e.g. [79,80]), and this may have led Cools *et al*. [9] to overestimate the frequency of both conflicts (1711 conflicts over two months of observation on three groups composed, respectively, by seven, seven and six dogs) and reconciliations (*N* = 606), compared with our study. Even though the factors affecting the occurrence of reconciliation in dogs are still unclear, it is interesting to note that, in both studies, reconciliation tendencies were markedly lower than any reported in wolf studies. Indeed, where wolves engage in conflict management, dogs seem to avoid each other. This is in line with results from Cools *et al*. [9], who found that, when a conflict was not immediately followed by reconciliation, opponents usually avoided each other for some time [9]. Actually, this result suggests that dogs may use a different PC strategy from that used by wolves. Interestingly this ‘avoidance strategy' has also been found in dogs, but not in wolves in a feeding context in that dogs are less tolerant of proximity during feeding on a monopolizable food source than wolves, tending to avoid conflicts by maintaining distance rather than using communication to negotiate access as wolves do [81,82].

Taken together current results on PC behaviour and results on food tolerance with similarly raised captive wolves and dogs suggest that wolves may use communication (including aggressive displays) with each other more frequently to negotiate and resolve conflict than dogs, which only engage in reconciliation under specific conditions (e.g. if relatives are involved).

The difference in the social ecology of dogs compared with wolves may explain the difference in their tendency to reconcile. In fact, dogs appear to depend mostly on scavenging rather than group hunting and rarely show cooperative breeding (e.g. provisioning of pups by all pack members). This reduced dependence on pack members may have relaxed the selection for communicative complexity [42,73–75], resulting in less frequent communicative threats, more intense physical aggression when conflicts occur and avoidance strategies remaining as the main alternative.

### 4.2. Factors affecting reconciliation in timber wolves

As regards the characteristics of the conflict itself, variation in wolf reconciliation was mainly influenced by two factors: the context in which the conflict occurred and the intensity of the aggression. According to our predictions and in line with previous findings in wolves [43] and other species (e.g. [83–87]), reconciliation occurred more often and was more intense (showing multiple affiliative behaviours) in the non-feeding contexts than during feeding sessions. In contrast to our prediction, the latency and duration of reconciliation were not influenced by the context. Overall, because in the feeding context animals are busy eating, opponents often do not engage in reconciliation, but if they do they invest the same amount of time in the interaction as in the non-feeding context. Nevertheless, during feeding they just reconcile in a simple way. We also found that victims were more likely to initiate reconciliation than aggressors in the non-feeding context, whereas, after conflict over food, both victim and aggressor initiated friendly reunions at the same frequency. Indeed, in both non-feeding and feeding contexts victims were subordinate to the aggressors in most of the conflicts (non-feeding context: 86% of conflicts; feeding context: 89.9% of conflicts). Therefore, it may be suggested that, during feeding, both subordinate victims and dominant aggressors perceive a stronger advantage from the maintenance of a peaceful relationship, because restoring the relationship quickly can allow them to return to feeding, reducing the risk of further interruptions (see [52]).

The intensity of aggression affected the likelihood of reconciliation occurring. According to our prediction, high-intensity aggressions (i.e. conflict with physical contact) were followed by PC affiliative interactions more often than low-intensity aggressions, but, contrary to our expectations, the latency of reconciliation did not differ between them. The intensity of reconciliation also varied according to the intensity of the preceding conflict, such that reconciliation was more intense after higher intensity conflicts. This suggests that relationships between opponents may be increasingly disturbed with increasing conflict intensity and that the occurrence of reconciliation and its intensity may help to restore the disrupted relationships.

Both the security of the relationship and the competitive difference between opponents influenced some aspects of reconciliation. In particular, the security of the relationship (measured by the asymmetry in the exchange of affiliative behaviours) affected the duration of reconciliation. Our results showed that longer PC affiliative interactions occurred in dyads with a low relationship security (i.e. where the affiliative asymmetry was high and biased towards the aggressor). Therefore, apparently and in contrast to our prediction and previous findings [53,57], the duration of reconciliation increases with a decrease in the security of the opponent's affiliative relationship. Nevertheless, because victims were responsible for the initiation of reconciliation after the majority of conflicts, they indeed engaged in longer reconciliations with those aggressors from whom they have a higher probability of being reciprocated.

Furthermore, dyads in which the asymmetry of aggression was more heavily biased towards the aggressor reconciled with more intense interactions. This result may be explained by the rank relationship between the two opponents; in fact, the intensity of reconciliation may increase with the increasing risk of re-aggression by the dominant aggressor towards the subordinate victim, thus with a higher difference in the competitive abilities of the two opponents. Indeed, we found that the higher the rank distances between opponents, the more likely the occurrence of reconciliation. Since in most of the conflicts the victim was subordinate to the aggressor (91.5%), the higher occurrence of reconciliation between opponents more distant in rank may be directly linked to the potentially higher costs for the subordinate victims in case of a renewed aggression, and thus the higher benefits of restoring their relationship with the dominant aggressors.

Overall, we found evidence that conflicts in wolves may disrupt the relationship between opponents and that the variation of reconciliation is a consequence of a compromise between costs and benefits of the interaction and for the particular relationship of the two opponents. Our results are in line with the suggestion that reconciliation functions to repair relationships because it reduces the probability of renewed aggression (*relationships repair hypothesis*, e.g. [20,84,88–91]; see also [43,92]).

## 5. Conclusion

In conclusion, the results of our dog–wolf comparison suggest that wolves are more communicative in their social interactions after conflicts, seeking their partner out to re-establish the relationship, whereas dogs appear to adopt an avoidance strategy. This is in line with the avoidance observed in a feeding context when potential for conflicts is high [81] and with the different social ecology of the two species [42]. Nevertheless, contrasting results found in other studies highlight the need for further investigations in order to clarify which factors may promote and modulate reconciliation in domestic dogs. Contrary to dogs, we confirmed the occurrence of reconciliation in wolves, providing, for the first time, new insight into the factors affecting its variation.

## Supplementary Material

Cafazzo_et_al_S1_S2_tables_ESM.doc

## Supplementary Material

Cafazzo_et_al_David_Score_Calculation_ESM

## Supplementary Material

Cafazzo_et_al_Data_used_for_analyses_ESM

## Footnotes

1 We recorded fewer PC–MC pairs than the total number of aggressive interactions observed for two main reasons. First, most of the aggression came from data recorded during the ‘focal animal sampling’, which was used to independently assess the relationship between the pack animals. Second, during the ‘behavioural sampling’ periods, it was not always possible to follow the victim for the entire 10 min period, because the subjects sometimes moved fast and went out of sight. Furthermore, for the same reasons, it was not always possible to collect the MC for each PC period collected. As a result of all these factors, the final number of PC–MC was lower than the total number of aggressive interactions recorded.

## Data accessibility

The datasets supporting this article have been uploaded as part of the electronic supplementary material.
