# Supplementary material for: The effect of domestication on post-conflict management: wolves reconcile while dogs avoid each other
Source: R Soc Open Sci. 2018 Jul 4;5(7):171553. doi: 10.1098/rsos.171553 (PMC6083655; doi:10.1098/rsos.171553)
Supplement: Cafazzo_et_al_S1_S2_tables_ESM.doc [file rsos171553supp1.docx]

S1 Table. Ethogram used to collect data during the Focal animal sampling sessions.

| **Category** | **Behaviours** | **Description** |
| --- | --- | --- |
| **Affiliative behaviours** | | |
|  | Grooming | To nip, lick or scratch the fur or skin occasionally the neck of the receiver's |
|  | Inspection | To interact friendly, stand next to each other, rubbing against each other side by side, smelling each other, putting heads together and licking, sniffing and so on |
|  | Lie friendly | To lie on the back, tail-wagging, maybe kicking with the foreleg against or toward another subject sometimes with open mouth |
|  | Stand friendly | The subject stands with tail horizontal to or below the plane of the back, wagging it, ears pointed forward, while another is approaching it or orienting/looking towards it |
|  | Body contact | Two subjects stay (for at least 10 s) with at least a part of their bodies in contact and in a relaxed position |
|  | Social sniff | To sniff another’s body part except its anogenital area |
|  | Body rubbing | To rub ones body against any part of the receiver’s |
|  | Approach friendly | To approach another subject within one body length remaining within that distance for at least 5 seconds. The approach is characterized by the subject holding the tail perpendicular to or below the plane of the back and wagging it |
|  | Nose touch | Brief nose touch by one wolf to the face or body of another wolf; no tail wag, ears may be flattened |
|  | Muzzle licking | To lick the other's lips and nose. The tail is relaxed and below the plane of the back |
| **Dominance behaviours** | | |
|  | Stand tall | Subject traightens up to full height, with a rigid posture and tail, may include raised hackles, ears erect and tail perpendicular or above the back |
|  | Stand over | To stand over another's body, with all four paws on the ground and the tail above the plane of the back. The receiver may have either the whole body or just the forepaws under the actors’ belly/side |
|  | Paw on | To place one or both forepaws on the other’s back |
|  | Ride up | To mount another one from behind or from the side, exhibiting a thrusting motion |
|  | Head on | The subject approaches another’s shoulder/back with the tail above the plane of the back and puts its head on it. Most of times formation looks like a capital “T” |
|  | Muzzle bite | To grab the muzzle of another subject softly |
|  | Approach dominant | To approach another subject within one body length for at least 5 seconds, with the tail perpendicular or above the plane of the back and the ears erect and pointed forward |
| **Submissive behaviours** | | |
|  | Crouch | Lowering the head, sometimes bending the legs, arching the back, lowering the tail between the hindlegs, and avoiding eye contact |
|  | Passive submission | To lie on the back showing the stomach and holding the tail between the legs. The ears are held back and close to the head and the subject raises a hind leg for inguinal presentation |
|  | Active submission | The subject has its tail tucked between the hind legs sometimes wagging it while he is in a crouched position (with hindquarters lowered) and may attempt to paw and lick the side of actors’/aggressor’s muzzle. The behaviour may include urination |
|  | Withdrawing | The subject withdraws from another moving away slowly in the opposite direction, displaying a submissive posture. It occurs when a subject has been threatened or attacked by another, or a fight has taken place |
|  | Flee | To run away from another with tail tucked between the legs and body ducked. It occurs when a subject has been threatened or attacked by another, or after a fight |
|  | Avoidance | In response to another reducing the distance towards it, the subject moves away displaying a submissive posture. The subject may also look at the individual he is trying to avoid |
|  | Approach submissive | To slowly approach another within one body length remaining within that distance for at least 5 seconds. The approach is characterized by a ducked posture and tail between the legs. Subject can also be moving in a wavy line and in a hesitant (stop-start) manner |
| **Aggressive behaviours** | | |
|  | Threat | Subject orients towards another performing one or more of the following: staring at, curling of the lips, baring of the canines, raising the hackles, snarling, growling, and barking, sometimes with the tail perpendicular or above the back |
|  | Attack | Running into or jumping onto another with tail, ears and sometimes hackles up, often with bites at the neck |
|  | Knock down | To strike another subject sharply with the chest or shoulder so that the other falls to the ground |
|  | Stand over aggressive | To overwhelm another subject, stopping on it in a stand over position with the tail above the plane of the back, growling and showing piloerection |
|  | Pin | To grab another at the neck or at the muzzle, forcing it down to the ground and holding it there |
|  | Fight | The subject and the receiver engage in reciprocal biting and aggressive physical contact |
|  | Chase | A subject runs after a conspecific, exhibiting threatening behaviours (see ‘threat’ above) |
|  | Jaw spar | Two subjects "fencing" with open jaws |
|  | Snapping | To snap teeth into the air, noisily |
|  | Bite | Bite a conspecific, without inhibition, with enough pressure to cause potential injury |
|  | Submissive aggression | The subject is in a crouched posture, tail tucked in and sometimes with a paw up but shows teeth whilst having the corners of the mouth pushed forward, often accompanied by snapping, pushing the other away, and/or barking |

S2 table. Ethogram used to code the PC/MC videos collected during the Behavioural sampling sessions.

| **Category** | **Behaviours** | **Description** |
| --- | --- | --- |
| **Affiliative behaviours** | | |
|  | Grooming | To nip, lick or scratch the fur or skin occasionally the neck of the receiver's |
|  | Inspection | To interact friendly, stand next to each other, rubbing against each other side by side, smelling each other, putting heads together and licking, sniffing and so on |
|  | Play invitation | Two or more subjects engage in motor patterns such as bite, chase, run around one another, kick, jump and maybe snap or bite without enough pressure to cause injury. These patterns can be typical of ‘serious’ functional contexts (e.g. agonistic, anti-predatory) but in a different manner. In fact, playful behaviours are often exaggerated, reordered, incomplete, brief, repeated, varied in sequence and inhibited. A play bout is considered over after 30 seconds of non-play. |
|  | Play | Two or more subjects engage in motor patterns such as bite, chase, run around one another, kick, jump and maybe snap or bite without enough pressure to cause injury. A play bout is considered over after 30 seconds of non-play |
|  | Lie friendly | To lie on the back, tail-wagging, maybe kicking with the foreleg against or toward another subject sometimes with open mouth |
|  | Stand friendly | The subject stands with tail horizontal to or below the plane of the back, wagging it, ears pointed forward, while another is approaching it or orienting/looking towards it |
|  | Body contact | Two subjects stay (for at least 10 s) with at least a part of their bodies in contact and in a relaxed position |
|  | Social sniff | To sniff another’s body part except its anogenital area |
|  | Body rubbing | To rub ones body against any part of the receiver’s |
|  | Approach friendly | To approach another subject within one body length remaining within that distance for at least 5 seconds. The approach is characterized by the subject holding the tail perpendicular to or below the plane of the back and wagging it |
|  | Nose touch | Brief nose touch by one wolf to the face or body of another wolf; no tail wag, ears may be flattened |
|  | Muzzle licking | To lick the other's lips and nose. The tail is relaxed and below the plane of the back |
| **Submissive behaviours** | | |
|  | Crouch | Lowering the head, sometimes bending the legs, arching the back, lowering the tail between the hindlegs, and avoiding eye contact |
|  | Passive submission | To lie on the back showing the stomach and holding the tail between the legs. The ears are held back and close to the head and the subject raises a hind leg for inguinal presentation |
|  | Active submission | The subject has its tail tucked between the hind legs sometimes wagging it while he is in a crouched position (with hindquarters lowered) and may attempt to paw and lick the side of actors’/aggressor’s muzzle. The behaviour may include urination |
|  | Withdrawing | The subject withdraws from another moving away slowly in the opposite direction, displaying a submissive posture. It occurs when a subject has been threatened or attacked by another, or a fight has taken place |
|  | Flee | To run away from another with tail tucked between the legs and body ducked. It occurs when a subject has been threatened or attacked by another, or after a fight |
|  | Avoidance | In response to another reducing the distance towards it, the subject moves away displaying a submissive posture. The subject may also look at the individual he is trying to avoid |
|  | Approach submissive | To slowly approach another within one body length remaining within that distance for at least 5 seconds. The approach is characterized by a ducked posture and tail between the legs. Subject can also be moving in a wavy line and in a hesitant (stop-start) manner |
| **Aggressive behaviours** | | |
|  | Threat | Subject orients towards another performing one or more of the following: staring at, curling of the lips, baring of the canines, raising the hackles, snarling, growling, and barking, sometimes with the tail perpendicular or above the back |
|  | Attack | Running into or jumping onto another with tail, ears and sometimes hackles up, often with bites at the neck |
|  | Knock down | To strike another subject sharply with the chest or shoulder so that the other falls to the ground |
|  | Stand over aggressive | To overwhelm another subject, stopping on it in a stand over position with the tail above the plane of the back, growling and showing piloerection |
|  | Pin | To grab another at the neck or at the muzzle, forcing it down to the ground and holding it there |
|  | Fight | The subject and the receiver engage in reciprocal biting and aggressive physical contact |
|  | Chase | A subject runs after a conspecific, exhibiting threatening behaviours (see ‘threat’ above) |
|  | Jaw spar | Two subjects "fencing" with open jaws |
|  | Snapping | To snap teeth into the air, noisily |
|  | Bite | Bite a conspecific, without inhibition, with enough pressure to cause potential injury |
|  | Submissive aggression | The subject is in a crouched posture, tail tucked in and sometimes with a paw up but shows teeth whilst having the corners of the mouth pushed forward, often accompanied by snapping, pushing the other away, and/or barking |
| **Proximity** |  |  |
|  | Close proximity | The victim is within three body length from former aggressor. |
