# Supplementary material for: The effect of domestication on post-conflict management: wolves reconcile while dogs avoid each other
Source: R Soc Open Sci. 2018 Jul 4;5(7):171553. doi: 10.1098/rsos.171553 (PMC6083655; doi:10.1098/rsos.171553)
Supplement: Cafazzo_et_al_David_Score_Calculation_ESM [file rsos171553supp2.docx]

**David’s score calculation**

Rank relationships (i.e. who was dominant and who submissive in each dyad), for all packs, were determined on the base of the frequency and direction of submissive interactions. An individual was considered to be the winner of an agonistic interaction when it received a submissive display.

In order to establish each individual’s rank position for all packs, we used the David’s score (DS), which has been found to be a very appropriate dominance ranking index (Gammell et al. 2003).

The proportion of wins by individual *i* in his interactions with another individual *j* (*Pij*) was the number of times that *i* defeats *j* (α*ij*) (i.e., the number of times it received a submissive display) divided by the total number of interactions between *i* and *j* (*nij*), i.e. *Pij*=α*ij*/*nij*.

The proportion of losses by *i* in interactions with *j*, *Pji*=1–*Pij*. If *nij*=0 then *Pij*=0 and *Pji*=0 (David 1988; de Vries 1998).

DS for each member, *i*, of a group was calculated with the formula:

DS=*w*+*w*2*l*-*l*2

where, *w* represents the sum of *i*’s *Pij* values, *w*2 represents the summed *w* values (weighted by the appropriate *Pij* values, see below) of those individuals with which *i* interacted, *l* represents the sum of *i*’s *Pji* values and *l*2 represents the summed *l* values (weighted by the appropriate *Pji* values) of those individuals with which *i* interacted (David 1988, page 108; de Vries 1998).
